# Use of prescribed analgesics before and after a standardised chiropractic care programme among patients with lumbar spinal stenosis: a nationwide cohort study

**DOI:** 10.1186/s12998-026-00639-x

**Published:** 2026-04-14

**Authors:** Rikke K. Jensen, Mathias Bech, Christian V. Skovsgaard, Jesper Ryg, Jan Hartvigsen, Melker S. Johansson

**Affiliations:** 1 Center for Muscle and Joint Health, Department of Sports Science and Clinical Biomechanics, University of Southern Denmark, Odense, Denmark; 2 Chiropractic Knowledge Hub, Odense, Denmark; 3 Spine Center of Southern Denmark, University Hospital of Southern Denmark, Sønderborg, Denmark; 4 Danish Centre for Health Economics (DaCHE), Department of Public Health, University of Southern Denmark, Odense, Denmark; 5 Department of Clinical Medicine, University of Copenhagen, Copenhagen, Denmark; 6 Geriatric Research and Clinical Evidence (GRACE), Department of Internal Medicine Geriatric Section, Copenhagen University Hospital - Herlev and Gentofte Hospital, Copenhagen, Denmark; 7 Geriatric Research Unit, Department of Clinical Research, University of Southern Denmark, Odense, Denmark; 8 Research Unit of General Practice, Department of Public Health, University of Southern Denmark, Odense, Denmark

**Keywords:** Lumbar spinal stenosis, Analgesics, Opioids, Gabapentinoids, Chiropractic care, Registry study, Prescribing patterns, Prognostic factors

## Abstract

**Background:**

Patients with degenerative lumbar spinal stenosis (LSS) commonly use analgesics despite not being recommended by clinical guidelines. However, detailed knowledge of utilisation patterns is limited. This study aimed to describe prescribed analgesic use before, during, and after enrolment in a standardised chiropractic care programme, and to examine associations between patient characteristics and continued analgesic use after the programme.

**Methods:**

This cohort study included patients with LSS enrolled in the chiropractic care programme between 1 April 2017 and 31 December 2022, identified via the Danish National Health Service Register. The programme provides guideline-based non-pharmacological care, structured follow-up, and coordination with the patient's general practitioner. Data on dispensed analgesics were retrieved from the Danish National Prescription Registry. Quarterly prevalence of analgesic use (≥ 1 dispensed prescriptions) and defined daily doses per 1000 individuals were calculated for the year before enrolment, 3 months during, and the year after the programme. The distribution of use was assessed using Lorenz curves and Gini coefficients. Associations between patient characteristics and continued analgesic use (≥ 1 dispensed prescriptions in ≥ 2 of 4 consecutive quarters) after the programme among existing users were examined using robust Poisson regression.

**Results:**

Of the 7294 included patients, 45% used analgesics in the year before enrolment. Use of paracetamol, non-steroidal anti-inflammatory drugs (NSAID), opioids, and gabapentinoids increased in the year before enrolment, peaked during the programme, and generally declined after. Ten percent of analgesic users accounted for approximately 43%, 49%, 68%, and 57% of the total use of paracetamol, NSAIDs, opioids, and gabapentinoids, respectively. Female sex, having one comorbidity, and pre-existing use of paracetamol, opioid, or gabapentinoid were associated with a 5–16% higher risk of continued use, whereas long-cycle higher education was associated with a 13% lower risk.

**Conclusions:**

Analgesic use increased before enrolment, peaked during the programme, and then declined after. A small proportion of analgesic users accounted for a large share of the total use. Sociodemographic and health-related factors, and pre-existing analgesic use were associated with continued use. These findings highlight the complexity of analgesic use in this population and emphasise the need for deprescribing strategies that take this complexity into account.

**Supplementary Information:**

The online version contains supplementary material available at 10.1186/s12998-026-00639-x.

## Introduction

Lumbar spinal stenosis (LSS) is caused by degenerative changes in the lumbar spine that result in narrowing of the spinal canals [1, 2]. In some individuals, progressive compression of the neurovascular structures can lead to leg pain and neurological symptoms, typically described as neurogenic claudication [1]. LSS symptoms contribute to reduced physical activity, limitations in performing activities of daily living, and poor quality of life [1]. Due to the degenerative nature of the condition, its prevalence increases with age [3]. Accordingly, LSS is among the most common causes of pain and disability in older adults and is the leading indication for spinal surgery in individuals over the age of 65 years [4]. With an ageing population, the burden of LSS is expected to grow even further, both for individuals and healthcare systems.

Clinical guidelines discourage the use of analgesics (e.g., paracetamol, non-steroidal anti-inflammatory drugs (NSAIDs), gabapentinoids, and opioids) for managing LSS-related symptoms due to the limited evidence supporting meaningful benefit. Furthermore, the typical clinical presentation of LSS, in which symptoms are intermittent and activity-related, may not always justify the prescription of analgesics. Instead, clinical guidelines recommend multimodal treatment programmes including patient education, exercise, and manual therapy [5, 6]. To support guideline-recommended treatment, the Danish Chiropractic Association and Danish Regions have negotiated, within the framework of a collective agreement, increased reimbursement for patients receiving a standardised chiropractic care programme. The programme outlines a management structure and a care pathway for patients with LSS, including scheduled follow-up consultations at 4 weeks and 3 months to monitor symptom progression, reassess the diagnosis and treatment plan, and inform the patient’s general practitioner (GP).

The recommendations against analgesic use are based on a lack of evidence of effectiveness for some analgesics, evidence of no effectiveness for others, and the risk of significant adverse events especially among older people with complex health problems and multimorbidity [5–7]. For example, long-term paracetamol use may cause liver damage [8, 9]; NSAIDs are associated with an increased risk of gastrointestinal complications, renal dysfunction, and cardiovascular events [10–12]; and opioids pose risks of addiction, falls, and increased mortality [13–17]. These risks underscore the importance of patient safety when considering pharmacological treatment options for LSS. Careful evaluation of analgesic use is essential to avoid exposing patients to unnecessary harm, particularly when safer, guideline-consistent alternatives are available.

Despite these risks and guideline recommendations, analgesics are commonly used by patients with LSS. A Swedish study reported annual opioid prescriptions in 67% of patients with LSS [18]. Similar trends are seen in other degenerative joint conditions, such as osteoarthritis, where up to 46% of patients in primary care used any analgesics [19] with 19% using opioids specifically [20]. Notably, a small group of patients accounted for a large share of total opioid use, and the use increased in the year preceding participation in an exercise and education programme and declined afterwards [21].

However, detailed knowledge of utilisation patterns among patients with LSS is limited. Therefore, this study aims to investigate utilisation patterns of prescribed analgesics before, during, and after enrolment in a standardised chiropractic care programme for patients with LSS, and to examine the association between patient characteristics and continued analgesic use following the care programme.

## Methods

The reporting of the current study adheres to the Strengthening the Reporting of Observational Studies in Epidemiology (STROBE) statement [22].

### Data sources

This observational cohort study is based on individual-level data from Danish national registries linked using the national civil registration number.

Chiropractic health insurance service codes were retrieved from the Danish National Health Insurance Service Register [23], which specifically identify patients enrolled in the standardised chiropractic care programme for LSS at clinics operating under a reimbursement agreement [24]. Approximately 94% of chiropractic clinics in Denmark operate under this collective agreement [25].

Dispensed prescriptions recorded in the Danish National Prescription Registry [26], which includes all drugs dispensed at pharmacies in Denmark, were used as a proxy for analgesic use. In the Danish healthcare system, consultations with GP are free for patients, although co-payments are required for prescription medications. Doctors, including GPs, are responsible for issuing all prescriptions in Denmark [27], whereas chiropractors do not prescribe.

Diagnostic codes for i) comorbidities, ii) previous LSS diagnoses, and iii) prior decompression surgery for LSS, each within the 5 years preceding the index date, were obtained from the Danish National Patient Register [28, 29], which includes data on all contacts with private and public hospitals in Denmark. Diagnostic codes are based on the International Classification of Diseases (ICD-10) [30]. These variables were not used to determine eligibility for the chiropractic care programme but were collected to describe the study population and to serve as candidate prognostic factors.

Demographic data on age, sex, cohabitation status, geographical region in Denmark (North Denmark Region, Central Denmark Region, Region of Southern Denmark, Region Zealand, and Capital Region of Denmark), level of education, income, and employment status were retrieved from the Danish Civil Registration System [31], the Population Education Registry [32], and the Income Registry [33].

### Study population

Patients were identified through chiropractic service codes in the Danish National Health Insurance Service Register, and included if they were aged 18 years or older, and enrolled in a standardised chiropractic care programme for LSS between 1 April 2017 and 31 December 2022. The first consultation (index date) was identified using the health insurance service codes ‘Initial assessment for LSS’ (code 1060), and ‘Assessment for suspected LSS in patients undergoing current treatment’ (code 1061) [24]. If patients had more than one code for an LSS programme registered, only the first code was used. Enrolment in the care programme required clinical signs and symptoms of LSS, as assessed by a chiropractor, including leg pain, and reduced walking distance, as described in the collective agreement [24].

As pain due to cancer is a clinical indication for opioid use, patients were excluded if a cancer diagnosis had been recorded within 5 years before the index date (ICD-10 code C00-97, excluding C43: malignant melanoma of the skin). Study participants were hence required to have register-data coverage 5 years before the index date.

### Variables

The outcomes were i) the prevalence of one or more dispensed prescriptions within one quarter (90 days), ii) total number of dispensed defined daily doses (DDDs) per quarter, and iii) continued analgesic use in the year following the care programme. Analgesic use included oral and transdermal routes of administration of paracetamol, NSAIDs, opioids, gabapentinoids, serotonin-norepinephrine reuptake inhibitors (SNRIs), tricyclic antidepressants (TCAs), and muscle relaxants (Supplementary Table S1).

A DDD is a standardised measure representing the assumed average maintenance dose per day for a drug when used for its main indication in adults [34]. DDDs are commonly used in pharmacoepidemiology to quantify and compare drug utilisation across populations, independent of variations in prescribed dose or package size. Prevalent analgesic use was defined as one or more dispensed prescription within at least two of the four quarters before the index date. Continued analgesic use was defined as one or more dispensed prescription in at least two of the four quarters in the year after the care programme, among those with pre-existing use. This definition was applied separately to paracetamol, NSAIDs, opioids, and gabapentinoids, as well as to overall use (i.e., use of at least one of the four analgesic classes), as these have previously been identified as the most commonly used analgesics in primary care populations [21].

Candidate prognostic factors for continued analgesic use included registered biological sex, age at the index date, highest attained education (classified as primary school, upper secondary or vocational education, and short-, medium-, or long-cycle higher education) [32], annual income (grouped into quartiles), retirement status, cohabitation status (cohabiting vs non-cohabiting), number of comorbidities from the Charlson Comorbidity Index based on ICD-10 codes [35, 36], previous LSS diagnosis in secondary care within 5 years before the index date, previous decompression surgery within 5 years before the index date (Supplementary Table S2), and use of either paracetamol, NSAIDs, opioids, or gabapentinoids in the year before the index date, defined as one or more dispensed prescription within at least two of the four quarters before index date.

### Statistical analysis

Patient characteristics were described using frequencies with percentages and medians with first and third quartiles, as appropriate, overall and stratified by analgesic use status the year before index date (≥ 1 dispensed prescription within ≥ 2 of the 4 quarters before index date). For descriptive comparisons between analgesic users and non-users, continuous variables were compared using the Wilcoxon rank-sum test and categorical variables using Pearson’s Chi-squared test. As these analyses were exploratory and descriptive, exact p-values are not reported.

For any analgesic use and for each class (i.e., paracetamol, NSAIDs, opioids, gabapentinoids, SNRIs, TCAs, and muscle relaxants), the quarterly prevalence and the total DDDs dispensed per quarter per 1,000 individuals were calculated. To investigate temporal trends in dispensing patterns, a sensitivity analysis was conducted by stratifying the analyses by calendar year.

The study period was divided into 90-days intervals (quarters, Q) indexed as Q-4 to Q + 4, where Q0 represents the 3-month period after enrolment (day 0–day 90). The year before enrolment (day −360 to day −1) corresponds to Q-4 to Q-1, and the year after the end of the care programme (day 91–day 450) corresponds to Q + 1 to Q + 4.

The distribution of paracetamol, NSAID, opioid, and gabapentinoid use during the study period (i.e., Q-4 to Q + 4) among users was examined using Lorenz curves and Gini coefficients. The Gini coefficient (ranging from 0–1) quantifies inequality in the distribution of DDDs, with values closer to 1 indicating that a small proportion of patients accounts for a large proportion of the total DDDs.

Associations between candidate prognostic factors and continued use of paracetamol, NSAIDs, opioids, gabapentinoids, and overall use (any of these four analgesic classes) after the care programme were examined using Poisson regression with robust standard errors. Candidate prognostic factors were assessed both among pre-existing users overall and among users within each analgesic class. Relative risks (RRs) with 95% confidence intervals (CIs) for continued analgesic use were estimated from multivariable regression models including all candidate prognostic factors.

Given the differing mechanisms of action and safety profiles of the included analgesic classes, results are presented both separately by drug class and as a composite measure reflecting use of any included analgesic, without implying interchangeability in risks and benefits between medications.

All data analyses were performed using R version 4.3 (R Core Team, 2023) [37].

## Results

**Fig. 1:**
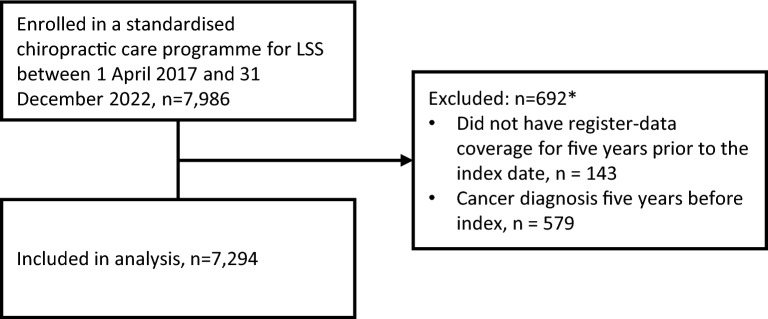
Study flow diagram. *Some individuals fulfilled both exclusion criteria

A total of 7,294 patients were included (Fig. 1). The median age was 71 years, and 52% were women (Table 1). Overall, 3,267 (45%) had one or more dispensed prescriptions for any type of analgesics in at least two of the four quarters during the year before the care programme. Demographic characteristics of analgesic users and non-users were broadly similar, although users were slightly more likely to be female, older, have lower educational level and income, live alone, be retired, have comorbidities, and to have received an LSS diagnosis or decompression surgery within the previous 5 years (Table 1).

**Table 1:** Characteristics of patients with LSS enrolled in a standardised chiropractic care programme, stratified by pre-programme analgesic user status

	Total	Analgesic users^a^	Analgesic non-users
	n (%)	n (%)	n (%)
Number of observations	7294	3267 (45)	4027 (55)
*Sex*			
Women	3698 (52)	1875 (59)	1823 (46)
Age in years, median (Q1, Q3)	71.2 (63.6, 77.1)	72.4 (65.5, 78.3)	70.2 (62.2, 76.0)
*Age group*			
< 50 yrs	362 (5)	112 (4)	250 (6)
50–59 yrs	834 (12)	317 (10)	517 (13)
60–69 yrs	2015 (28)	856 (27)	1159 (30)
70–79 yrs	2778 (39)	1293 (41)	1485 (38)
≥ 80 yrs	1104 (16)	594 (19)	510 (13)
*Level of education*^*b*^			
Primary school	1939 (27)	998 (31)	941 (24)
Upper secondary or vocational education	3310 (46)	1499 (46)	1811 (46)
Short-cycle higher education (< 3 yrs. beyond secondary school)	280 (4)	120 (4)	160 (4)
Medium-cycle higher education (3–4 yrs. beyond secondary school)	1,331 (18)	511 (16)	820 (21)
Long-cycle higher education or higher (≥ 5 yrs. beyond secondary school)	353 (5)	106 (3)	247 (6)
Annual income in EUR, median (Q1, Q3)	27,316 (21,005, 38,073)	25,480 (19,819, 34,287)	29,466 (21,986, 41,022)
*Income quartiles*			
Income Q1	1824 (25)	954 (29)	870 (22)
Income Q2	1823 (25)	925 (28)	898 (22)
Income Q3	1823 (25)	776 (24)	1047 (26)
Income Q4	1824 (25)	612 (19)	1212 (30)
Living alone	4455 (63)	1273 (40)	1365 (35)
Retired	4920 (67)	2386 (73)	2534 (63)
*Region of residence*			
North Region Denmark	413 (6)	215 (7)	198 (5)
Central Denmark Region	1637 (23)	712 (22)	925 (24)
Region of Southern Denmark	2460 (35)	1090 (34)	1370 (35)
Region Zealand	1192 (17)	559 (18)	633 (16)
Capital Region Denmark	413 (6)	215 (7)	198 (5)
*Comorbidities*^*c*^			
Chronic obstructive pulmonary disease	379 (5)	243 (7)	136 (3)
Cerebrovascular disease	327 (4)	169 (5)	158 (4)
Peripheral vascular disease	297 (4)	167 (5)	130 (3)
Congestive heart disease	172 (2)	93 (3)	79 (2)
Rheumatoid disease	167 (2)	124 (4)	43 (1)
*Number of comorbidities*			
0	5600 (77)	2293 (70)	3307 (82)
1	1295 (18)	732 (22)	563 (14)
≥ 2	399 (5)	242 (7)	157 (4)
LSS diagnosis in previous five yrs	720 (10)	499 (15)	221 (5)
Previous decompression surgery	254 (3)	189 (6)	65 (2)

Numbers are n (%) unless otherwise specified. EUR, euro; LSS, lumbar spinal stenosis; Q, quartile. a ≥ 1 dispensed prescription within ≥ 2 quarters of the year before index date (quarter Q-4 to Q-1); b Missing values 81 (1.1%); c Five most prevalent comorbidities/chronic diseases in Charlson comorbidity index based on ICD-10 codes. Statistically significant differences (p < 0.05) between users and non-users were observed for all variables based on Wilcoxon rank-sum test (continuous variables) and Pearson’s Chi-squared test (categorical variables)

**Fig. 2:**
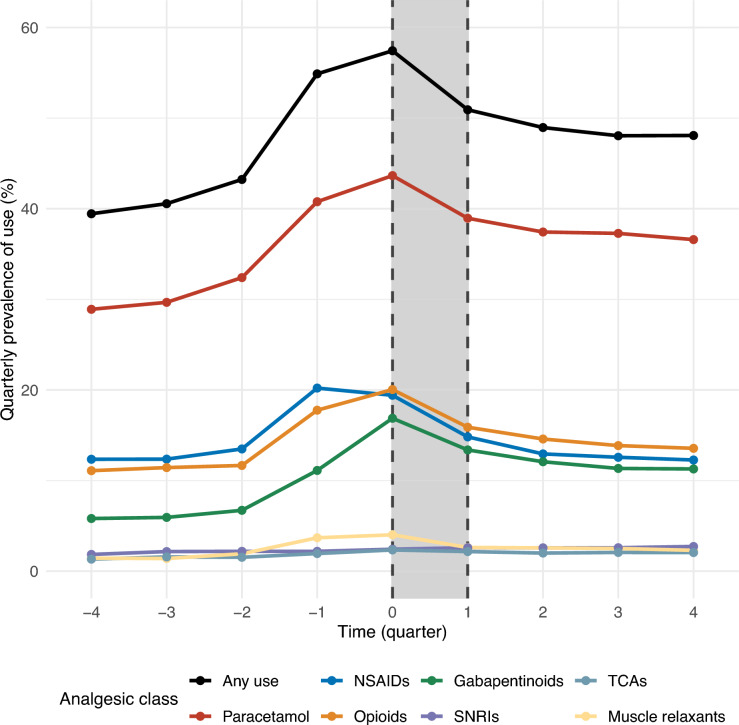
Quarterly prevalence of prescribed analgesics before, during, and after enrolment in a standardised chiropractic care programme. Quarterly prevalence of prescribed analgesics, overall and stratified by analgesic class, in 7,294 patients with LSS before, during, and after enrolment in a standardised chiropractic care programme. NSAID, non-steroidal anti-inflammatory drugs; SNRIs, serotonin-norepinephrine reuptake inhibitors; TCAs, tricyclic antidepressants. Q-4 corresponds to the first quarter of the year before index date, Q0 corresponds to the quarter after the index date, etc.

The prevalence of any analgesic use was 39% in the first quarter the year before the index date (Q-4), 57% during the care programme (Q0), and 48% in the last quarter of the year after the programme ended (Q + 4). In general, the quarterly prevalence of paracetamol, NSAID, opioid, and gabapentinoid use increased in the year before the index date and decreased after, especially during quarters Q-2 to Q + 2 (Fig. 2). In the first quarter of the year before enrolment (Q-4), the prevalence of paracetamol, NSAID, opioid, and gabapentinoid use was 29%, 12%, 11%, and 6%, respectively. During the quarter of the care programme (Q0), these figures had increased to 44%, 19%, 20%, and 17%. After the care programme (Q + 1 to Q + 2), a sustained decline was observed, and by Q + 4, the prevalence of NSAID and opioid use had decreased to 12% and 14%, close to the levels at Q-4, whereas paracetamol and gabapentinoid use remained higher at 37% and 11% compared with Q-4. The quarterly prevalence of SNRIs, TCAs, and muscle relaxants was 4% or less throughout the study period and remained relatively stable, except for a small increase followed by a decrease in the use of muscle relaxants around enrolment (Q-1 to Q1) (Fig. 2).

**Fig. 3:**
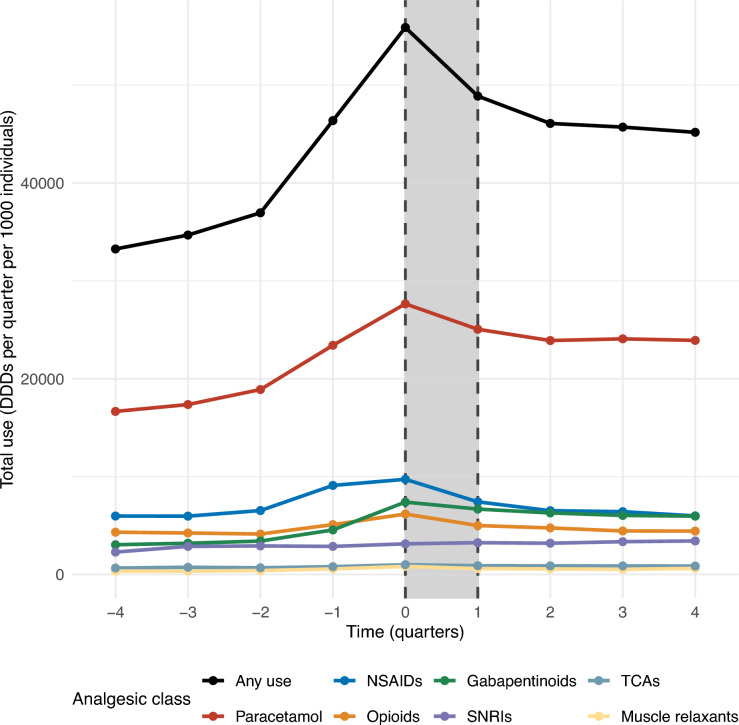
Quarterly DDDs of prescribed analgesics per 1,000 individuals before, during, and after a standardised chiropractic care programme. Quarterly DDDs of prescribed analgesics stratified by analgesic class per 1,000 individuals in 7,294 patients with LSS before, during, and after enrolment in a standardised chiropractic care programme. DDD, defined daily doses; NSAID, non-steroidal anti-inflammatory drugs; SNRIs, serotonin-norepinephrine reuptake inhibitors; TCAs, tricyclic antidepressants. Q-4 corresponds to the first quarter of the year before index date, Q0 corresponds to the quarter after the index date, etc.

During the year before the care programme (from Q-4 to Q-1), the use (DDDs per 1,000 individuals) of paracetamol, NSAIDs, and opioids increased by 41%, 52%, and 18%, with most of the increase occurring within 6 months before the programme. From before to after enrolment in the care programme (Q0 to Q1), the use of paracetamol, NSAIDs, and opioids decreased by 9%, 24%, and 19%. One year after the end of the care programme (Q + 4), paracetamol use remained 44% higher than one year before, while the use of NSAIDs and opioids had returned to approximately the same level as at Q-4 (Fig. 3).

Gabapentinoid use increased by 50% during the year before the programme, decreased slightly from Q0 to Q1 (by 10%), and remained 96% higher than at Q-4 one year after the programme ended. The use of SNRIs, TCAs, and muscle relaxants was low and remained relatively stable throughout the period. Figure 3 and Supplementary Table S3.

**Fig. 4:**
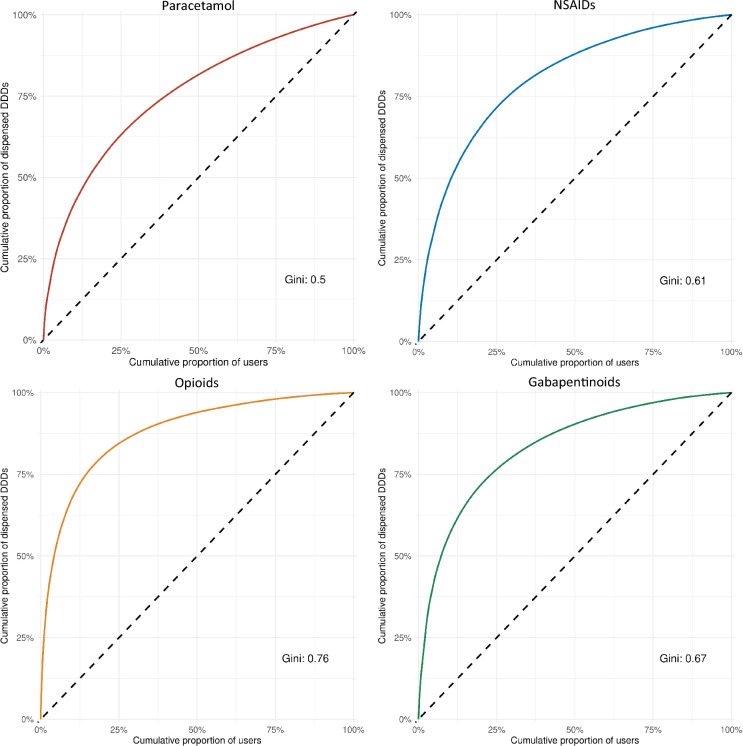
Lorenz curves illustrating the distribution of paracetamol, NSAIDs, opioids, and gabapentinoids prescriptions among individuals in a standardised chiropractic care programme. Lorenz curves illustrating the distribution of analgesic use in patients with LSS who dispensed one or more prescriptions during the study period. The Gini coefficient measures inequality in the distribution of defined daily doses (DDDs), reflecting how unevenly the drug is used across the patient population. A coefficient value of 0 indicates perfectly equal use, while 1 indicates highly unequal use

A small proportion of users of paracetamol, NSAIDs, opioid, and gabapentinoid accounted for a large share of the total DDDs dispensed during the study period. Specifically, 10% of users accounted for approximately 43%, 49%, 68%, and 57% of the total DDDs of paracetamol, NSAIDs, opioids, and gabapentinoids, respectively (Gini coefficients 0.50, 0.61, 0.76, and 0.67) (Fig. 4).

### Factors associated with continued analgesics use

Among patients with a pre-existing analgesic use (i.e., paracetamol, NSAIDs, opioids, or gabapentinoids), women (vs. men) and individuals with one comorbidity (vs. none) were 5% more likely to continue using analgesics in the year after the programme. Pre-existing paracetamol, opioid, and gabapentinoid use (vs. no use) were associated with a 16%, 10%, and 13% higher risk of continued analgesic use. In contrast, patients with a long-cycle higher education were 13% less likely to use analgesics after the programme compared to those with a primary school education (Table 2).

**Table 2:** Relative risks (RR) of continued analgesic use among existing users, overall and by analgesic class

Candidate prognostic factors	RR (95% CI)				
	Overall use n = 3139	Paracetamol use n = 2527	NSAID use n = 1004	Opioid use n = 850	Gabapentinoid use n = 467
*Sex*					
Women (vs. men)	**1.05 (1.01, 1.08)**	**1.06 (1.02, 1.11)**	0.91 (0.80, 1.02)	1.02 (0.91, 1.14)	1.07 (0.94, 1.21)
*Age group*					
< 50 yrs (ref.)	Ref	Ref	Ref	Ref	Ref
50–60 yrs	1.08 (0.95, 1.22)	1.06 (0.90, 1.24)	1.00 (0.78, 1.28)	0.99 (0.70, 1.40)	**0.68 (0.51, 0.90)**
60–70 yrs	1.08 (0.96, 1.22)	1.05 (0.90, 1.23)	0.88 (0.69, 1.14)	1.12 (0.80, 1.55)	0.89 (0.72, 1.11)
70–80 yrs	1.11 (0.98, 1.26)	1.08 (0.92, 1.28)	0.78 (0.58, 1.05)	1.04 (0.73, 1.49)	1.02 (0.77, 1.34)
≥ 80 yrs	1.13 (1.00, 1.29)	1.10 (0.93, 1.30)	0.72 (0.50, 1.03)	1.03 (0.71, 1.48)	1.07 (0.79, 1.43)
*Level of education*					
Primary school (ref.)	Ref	Ref	Ref	Ref	Ref
Upper secondary or vocational education	0.99 (0.96, 1.03)	0.99 (0.95, 1.03)	0.95 (0.82, 1.09)	0.93 (0.83, 1.05)	1.00 (0.87, 1.15)
Short-cycle higher education	0.93 (0.84, 1.03)	0.91 (0.80, 1.03)	0.74 (0.51, 1.08)	1.06 (0.81, 1.39)	**1.26 (1.03, 1.55)**
Medium-cycle higher education	1.00 (0.95, 1.05)	0.96 (0.90, 1.03)	0.99 (0.82, 1.18)	0.99 (0.85, 1.16)	1.03 (0.86, 1.24)
Long-cycle higher education	**0.87 (0.77, 0.99)**	**0.84 (0.71, 0.99)**	0.68 (0.46, 1.02)	0.84 (0.56, 1.24)	1.06 (0.75, 1.51)
*Yearly income (quartiles)*					
Income Q1 (ref.)	Ref	Ref	Ref	Ref	Ref
Income Q2	1.00 (0.96, 1.04)	1.03 (0.98, 1.08)	1.11 (0.93, 1.32)	1.01 (0.89, 1.16)	1.12 (0.95, 1.32)
Income Q3	0.98 (0.94, 1.03)	1.01 (0.95, 1.07)	1.09 (0.92, 1.31)	0.89 (0.76, 1.04)	1.15 (0.97, 1.36)
Income Q4	0.94 (0.89, 1.00)	0.97 (0.90, 1.05)	1.07 (0.88, 1.31)	**0.78 (0.62, 0.97)**	1.04 (0.84, 1.29)
*Non-cohabiting*					
Yes (vs. no)	1.00 (0.96, 1.04)	1.01 (0.96, 1.05)	1.00 (0.88, 1.13)	1.08 (0.97, 1.22)	1.00 (0.88, 1.14)
*Retired*					
Yes (vs. no)	1.01 (0.95, 1.08)	1.07 (0.99, 1.16)	1.06 (0.87, 1.28)	0.99 (0.81, 1.20)	0.83 (0.67, 1.04)
Number of comorbidities					
0 (ref.)	Ref	Ref	Ref	Ref	Ref
1	**1.05 (1.02, 1.09)**	1.04 (0.99, 1.08)	0.99 (0.85, 1.16)	1.05 (0.94, 1.18)	1.10 (0.96, 1.25)
≥ 2	1.03 (0.97, 1.09)	1.01 (0.94, 1.09)	0.85 (0.58, 1.24)	1.11 (0.95, 1.29)	1.07 (0.88, 1.31)
*LSS diagnosis in previous five yrs*					
Yes (vs. no)	1.03 (0.98, 1.07)	1.05 (0.99, 1.10)	1.04 (0.85, 1.26)	1.12 (0.99, 1.28)	0.93 (0.79, 1.08)
*Decompression surgery in previous five yrs*					
Yes (vs. no)	0.99 (0.92, 1.05)	0.99 (0.91, 1.07)	0.99 (0.74, 1.32)	**0.71 (0.56, 0.91)**	0.89 (0.72, 1.11)
*Pre-existing paracetamol use*					
Yes (vs. no)	**1.16 (1.11, 1.22)**	-	**1.21 (1.05, 1.39)**	**0.89 (0.79, 0.99)**	1.02 (0.89, 1.17)
*Pre-existing NSAID use*					
Yes (vs. no)	1.02 (0.99, 1.06)	1.03 (0.98, 1.08)	**-**	0.99 (0.87, 1.12)	**1.15 (1.01, 1.31)**
*Pre-existing opioid use*					
Yes (vs. no)	**1.10 (1.06, 1.13)**	**1.05 (1.01, 1.10)**	1.02 (0.88, 1.18)	-	1.10 (0.98, 1.24)
*Pre-existing gabapentinoid use*					
Yes (vs. no)	**1.13 (1.09, 1.17)**	**1.08 (1.03, 1.13)**	1.09 (0.91, 1.31)	**1.18 (1.05, 1.32)**	-

RRs were estimated using multivariable Poisson regression models with robust standard errors; all candidate prognostic factors were hence included in each model. Bold estimates indicates that the 95% CI does not contain the value 1. Number of comorbidities is based on ICD-10 codes from Charlson comorbidity index. Overall use is any of the four analgesics (i.e., paracetamol, NSAIDs, opioids, or gabapentinoids). CI, confidence interval; LSS, lumbar spinal stenosis; NSAID, non-steroidal anti-inflammatory drugs; Ref., reference category; RR, relative risk; Q, quartile: Yrs, years

Among those using paracetamol in the year before the programme, 80% continued to use it in the year after. Women were 6% more likely to continue use after the programme than men, while pre-existing opioid or gabapentinoid use (vs. no use) was associated with a 5% and 8% higher risk. Conversely, those with a long-cycle higher education (vs. primary school) were 16% less likely to continue using paracetamol after the programme (Table 2).

Among patients with pre-existing NSAID use, 52% continued the use after the programme. Pre-existing paracetamol use was associated with a 21% higher risk of continued NSAID use after the programme compared with those without a pre-existing paracetamol use (Table 2).

Of those using opioids before the programme, 63% continued use after the programme. Patients in the highest income quartile were 22% less likely to use opioids after the programme compared with those in the lowest quartile. Similarly, patients who had undergone decompression surgery within 5 years before the programme (vs. no previous LSS surgery) or pre-existing paracetamol use (vs. no use) were 29% and 11% less likely, respectively, to continue opioid use after the programme. In contrast, patients with pre-existing gabapentinoid use (vs. none) were 18% more likely to continue using opioids after the programme (Table 2).

Among patients using gabapentinoids before the programme, 70% continued to use it the year after the programme. Those aged 50–59 years were 32% less likely to continue using gabapentinoids compared with patients younger than 50 years. In contrast, patients with a short-cycle higher education (vs. primary school) or NSAID use (vs. no use) were 27% and 15% more likely, to continue gabapentinoid use after the programme (Table 2).

### Temporal trends

We found three dispensing patterns over time. First, DDDs for paracetamol and gabapentinoids increased steadily from 2017 to 2022. Similarly, DDDs of SNRIs showed a steady, though more modest, increase. Second, DDDs of NSAIDs remained relatively stable, with a peak around 2019 followed by a small decline by 2022. Similarly, muscle relaxants and TCAs also remained stable with only minor fluctuations across calendar years. Lastly, DDDs of opioids showed a consistent decline throughout the study period. (Supplementary Figure S1).

## Discussion

Despite guideline recommendations for non-pharmacological treatment, a large proportion (57%) of patients with LSS enrolled in a chiropractic care programme for LSS used prescription analgesics at enrolment, and nearly half (48%) continued to do so one year after the programme. Use of analgesics, particularly of paracetamol, NSAIDs, opioids, and gabapentinoids increased during the year before the programme, peaked during the programme, and generally declined after. Notably, a small proportion of users accounted for a large share of the total dispensed drugs, in particular opioids and gabapentinoids. Several patient characteristics were associated with continued use of analgesics.

The observed increase in analgesic use prior to and during the initiation of the chiropractic care programme may reflect symptom aggravation prompting patients to seek care. This pattern underscores the importance of monitoring medication use when patients enter non-pharmacological care. The observed decrease in analgesic use after the programme is likely a result of several factors. Firstly, although LSS is a degenerative condition, symptoms are known to fluctuate over time [38]. The increase in analgesic use before the programme may be followed by a decrease that reflects regression to the mean. Secondly, the observed changes in opioid and gabapentinoid use likely reflect broader temporal prescribing trends, including a general decrease in opioid use in Denmark between 2017 and 2023 [39], and an increase in gabapentinoid use [40], patterns also observed in a Swedish registry-based study of patients with neuropathic pain [18]. Finally, although the specific treatment modalities used (e.g., exercise, education, manual techniques) and frequency of treatment is not available in this dataset, some of the observed decrease in analgesics use might also be ascribed to the care programme. In line with this, a recent systematic review indicate that patients with LSS treated non-surgically experience a reduction in both pain and disability levels from baseline up to 12 months [41].

A small proportion of analgesic users accounted for a large proportion of total use of paracetamol, NSAIDs, opioids, and gabapentinoids. This was most evident for opioids and gabapentinoids, where 10% of patients accounted for 68% and 57% of total use. This is in line with findings from a prior study in a population of individuals with knee and hip osteoarthritis, where 10% of users accounted for 70% of opioid use [20]. This skewed distribution of use, highlight the need for clinical attention, monitoring of effect and adverse events, and tapering in case of inappropriate use.

We are not aware of previous studies that have examined prognostic factors for continued analgesics use in populations with LSS. In our study, female sex, having one comorbidity, and pre-existing overall analgesic use before enrolment in the programme were associated with increased risk of continued use after the programme, whereas those with a long-cycle higher education were less likely to continue. A few individual factors were associated with continued use of specific analgesic classes, but no clear pattern emerged. Pre-existing analgesic use was the most consistent finding across the four analgesic classes examined (paracetamol, NSAIDs, opioids, and gabapentinoids). Factors such as female sex and lower socioeconomic status have previously been linked to higher levels of pain and disability in populations with low back pain [42], suggesting that continued analgesic use may serve as a proxy for symptom severity [42].

Strengths of this study include its large, nationwide, register-based primary care cohort, with comprehensive linkages across national registries. The Danish National Prescription Registry includes all drugs dispensed at Danish pharmacies and are therefore considered both accurate and complete [43]. However, both paracetamol and NSAIDs are also available over the counter in Denmark, which is not captured in the prescription register. Nevertheless, over-the-counter sales of paracetamol and NSAIDs accounts for about 22% of total national sales [44]. These products are subject to restrictions, including small package sizes, and are not reimbursed. Consequently, individuals with frequent use of reimbursable analgesics (such as older individuals) have a practical and financial incentive to obtain them by prescription [43]. Therefore, the exclusion of over-the-counter analgesics from our data is unlikely to have significantly influenced the results although the total use is probably underestimated [45]. Also, the analyses included all prescribed opioids, encompassing both full and partial agonists (Supplementary Table S1). However, as the results are reported only at the class-level, it should be noted that safety profiles differ across specific opioid drugs.

A common limitation of studies using registry data is the uncertainty regarding whether dispensed prescriptions truly reflect actual medication use. A survey of 2410 participants found that a substantial proportion of individuals prescribed analgesics reported using lower doses than prescribed [46], indication a potential discrepancy between prescription and consumption. Additionally, the specific reason for taking the medication is not recorded. As LSS primarily affects older adults, they often have co-occurring degenerative conditions, such as hip and knee osteoarthritis, which are not included in the Charlson comorbidity index and, therefore, not registered as comorbidities in this study. Although analgesic use is relatively high in primary care populations with hip or knee osteoarthritis [21], such medications are generally not recommended for these conditions [47].

Another limitation is the potential lack of generalisability to all patients with LSS, particularly those managed outside chiropractic settings. Previous research has shown that patients with low back pain seen in general practice tend to be older, have a lower level of education, are more likely to be female, and generally present with more severe disease-related characteristics compared to those seeking chiropractic care [48]. Nevertheless, the results are considered generalisable to patients with LSS receiving chiropractic care, as this study includes a large, nationwide cohort with high coverage. The registers used in this study include all chiropractic clinics operating under a collective agreement with the Danish health authorities, which covers approximately 94% of all chiropractic clinics in Denmark [25], and chiropractors are well integrated into the Danish primary care system. However, we included all patients meeting the code-based criteria for enrolment in the chiropractic care programme and excluded repeated registrations within the study period by retaining only the earliest recorded programme code (since the introduction of the care programme) for each patient. We did not exclude patients who had received chiropractic care prior to the study period since reasons for seeking care or diagnosis given at contact was not coded. Consequently, some patients may have had previous treatment for LSS or other musculoskeletal conditions that could influence their symptom presentation or analgesic use.

Importantly, the high use of analgesics observed in this study is not consistent with recommendations from recent international clinical guidelines for the management of LSS, which continue to emphasise the importance of avoiding analgesic treatment with limited benefit and considerable risk [5, 6]. The findings highlight a need for ongoing evaluation of real-world prescribing patterns to ensure that analgesic use is appropriate and that patients receive optimal counselling, care, and support in line with evidence-based recommendations. From a patient safety perspective, the concentration of use among a small subset of patients raises concerns about potential overuse and adverse events. These results underscore the importance of targeted deprescribing strategies to support safer care for patients with LSS.

## Conclusions

In this nationwide, register-based study of patients with LSS enrolled in a standardised chiropractic care programme, more than half used analgesics, which is not consistent with national nor international guideline recommendations. A small proportion of analgesic users accounted for most dispensed drugs, particularly for opioids and gabapentinoids. Use of all analgesics decreased after the programme with only paracetamol and gabapentinoid not reaching before-programme levels. The observed changes in analgesic use are likely due to a combination of the care programme, the natural course of symptoms, and temporal trends in prescribing patterns for analgesics. Our findings underscore the complexity of analgesic use and the importance of individually targeted strategies to support appropriate prescribing and deprescribing in patients with LSS.

## Supplementary Information

Additional file1 (PDF 423 KB)

## Data Availability

The data supporting the results of this study are available from Statistics Denmark's Research Service, but there are restrictions on the availability of these data, which have been used under licence for the current study and are therefore not publicly available.

## Abbreviations

CI: Confidence interval
CPR: Civil registration numbers
DDD: Defined daily doses
GP: General practitioners
ICD-10: International classification of diseases 10th revision
LSS: Lumbar spinal stenosis
NSAID: Non-steroidal anti-inflammatory drugs
Q: Quartile
RR: Relative risk
SNRIs: Serotonin-norepinephrine reuptake inhibitors
STROBE: Strengthening the reporting of observational studies in epidemiology
TCAs: Tricyclic antidepressants
