# Supplementary material for: Use of prescribed analgesics before and after a standardised chiropractic care programme among patients with lumbar spinal stenosis: a nationwide cohort study
Source: Chiropr Man Therap. 2026 Apr 14;34:19. doi: 10.1186/s12998-026-00639-x (PMC13185251; doi:10.1186/s12998-026-00639-x)
Supplement: Supplementary file 1 — Additional file1 (PDF 423 KB) [file 12998_2026_639_MOESM1_ESM.pdf]

Supplementary file

Table S1 Overview of ATC-codes, analgesics, analgesic class, and route of administration of included analgesics

ATC	Analgesic	Analgesic class, sub-group	Adm. R
		**PARACETAMOL**	
N02BE01	Paracetamol	Paracetamol	O
		**NSAIDs**	
M01AA01	Phenylbutazone	NSAIDs, other	O
M01AB01	Indometacin	NSAIDs, other	O
M01AB02	Sulindac	NSAIDs, other	O
M01AB03	Tolmetin	NSAIDs, other	O
M01AB05	Diclofenac	NSAIDs, diclofenac	O
M01AB55	Diclofenac, combinations	NSAIDs, diclofenac	O
M01AB08	Etodolac	NSAIDs, etodolac	O
M01AB16	Aceclofenac	NSAIDs, other	O
M01AC01	Piroxicam	NSAIDs, other	O
M01AC02	Tenoxicam	NSAIDs, other	O
M01AC05	Lornoxicam	NSAIDs, other	O
M01AC06	Meloxicam	NSAIDs, other	O
M01AE01	Ibuprofen	NSAIDs, ibuprofen	O
M01AE02	Naproxen	NSAIDs, naproxen	O
M01AE03	Ketoprofen	NSAIDs, other	O
M01AE04	Fenoprofen	NSAIDs, other	O
M01AE09	Flurbiprofen	NSAIDs, other	O
M01AE11	Tiaprofenic acid	NSAIDs, other	O
M01AE14	Dexibuprofen	NSAIDs, other	O
M01AE17	Dexketoprofen	NSAIDs, other	O
M01AE52	Naproxen and esomeprazole	NSAIDs, naproxen	O
M01AG02	Tolfenamic acid	NSAIDs, other	O
M01AH01	Celecoxib	NSAIDs, coxibs	O
M01AH02	Rofecoxib	NSAIDs, coxibs	O
M01AH05	Etoricoxib	NSAIDs, coxibs	O
N02BA01	Acetylsalicylic acid (Aspirin, Idotyl, Magnyl)	NSAIDs, salicylic acid and derivatives	O
N02BA11	Diflunisal (Donobid)	NSAIDs, salicylic acid and derivatives	O
N02BA51	Acetylsalicylic acid, caffeine (500 + 50 mg, Migpriv, Treo, Triplo)	NSAIDs, salicylic acid and derivatives	O
		**OPIOIDS**	
N02AA01	Morphine	Opioid, morphine	O
N02AA03	Hydromorphone	Opioid, other	O
N02AA04	Nicomorphine	Opioid, other	O
N02AA05	Oxycodone	Opioid, oxycodone	O
N02AA55	Oxycodone and naloxone	Opioid, oxycodone	O
N02AB01	Ketobemidone	Opioid, other	O
N02AB02	Pethidine	Opioid, other	O

Table continued			
**ATC**	**Analgesic**	**Analgesic class, sub-group**	**Adm. R**
N02AB03	Fentanyl	Opioid, other	TD
N02AC04	Dextropropoxyphene	Opioid, other	O
N02AD01	Pentazocine	Opioid, other	O
N02AE01	Buprenorphine	Opioid, other	TD
N02AG02	Ketobemidone, antispasmodics	Opioid, other	O
N02AJ06	Codeine, paracetamol (30 + 500 mg; Pinex Comp/Citodon)	Opioid, codeine	O
N02AJ06	Codeine, paracetamol (30.6 + 500 mg; Kodipar)	Opioid, codeine	O
N02AJ06	Codeine, paracetamol (28.4 + 400 mg; Fortamol)	Opioid, codeine	O
N02AJ07	Codeine, acetylsalicylic acid (9.6 + 500 mg; Kodimagnyl)	Opioid, codeine	O
N02AJ07	Codeine, acetylsalicylic acid (10 + 500 mg; Codyl)	Opioid, codeine	O
N02BA75	Codeine, caffeine, propyphenazone, salicylamide, magnesium oxide (9.6 + 50 + 150 + 250 + 80 mg; Kodamid)	Opioid, codeine	O
R05DA04	Codeine	Opioid, codeine	O
N02AX02	Tramadol	Opioid, tramadol	O
N02AX06	Tapentadol	Opioid, other	O
		**GABAPENTINOIDS**	
N03AX12	Gabapentin	Gabapentinoids, gabapentin	O
N03AX16	Pregabalin	Gabapentinoids, pregabalin	O
		**SNRIs**	
N06AX16	Venlafaxine	SNRIs, venlafaxine	O
N06AX21	Duloxetine	SNRIs, duloxetine	O
		**TCAs**	
N06AA02	Imipramine	TCAs, imipramine	O
N06AA09	Amitriptyline	TCAs, amitriptyline	O
N06AA10	Nortriptyline	TCAs, nortriptyline	O
		**MUSCLE RELAXANTS**	
M03*			O
			O
			O

*Includes all ATC subgroups under M03. ATC, Anatomical Therapeutical Chemical code; Adm. R, route of administration; NSAIDs, non-steroidal anti-inflammatory drug; O, oral; SNRIs, serotonin–norepinephrine reuptake inhibitors; TCAs, tricyclic antidepressants; TD, transdermal.

Table S2 Surgical procedure codes used to identify patients who received decompression surgery for lumbar spinal stenosis in the previous five years

**Code**	**Diagnosis**
**Identification of all patients with surgery**	
**KABC36**	Decompression of lumbar nerve root
**KABC56**	Decompression of lumbar spinal canal and nerve root
**KABC66**	Decompression of lumbar spinal cord
**Supplementary procedures and diagnosis**	
**KNAG43, KNAG44, KNAG46**	Anterior fusion with internal fixation in the lumbar region
**KNAG63, KNAG64, KNAG66**	Posterior fusion without fixation in the lumbar region
**KNAG73, KNAG74, KNAG76**	Posterior fusion with fixation in the lumbar region
**Exclusion diagnoses**	
**DM41x**	Scoliosis
**DM43x**	Deformations (except DM431 spondylolisthesis)
**DM46x**	Inflammation and infection
**DM51x**	Disc herniation
**DM485x**	Low energy fracture
**DM49x**	Spinal disease from secondary disease (e.g. tuberculosis)
**DS32x**	Fractures in the spine and pelvic region
**DD32**	Dural tumours
**DC412x**	Spinal cancer

Procedure codes had to be in combination with a primary or secondary ICD-10 diagnostic code of spinal stenosis (DM48.0). Not registered as surgery for lumbar spinal stenosis if diagnoses indicated scoliosis, deformation, inflammation or infection, fracture, tumors, cancer, or spinal disease from a secondary disease (e.g., tuberculosis).

Table S3 Quarterly DDDs per 1,000 individuals among 7,294 patients with LSS enrolled in a standardised chiropractic care programme in Denmark from 1 April 2017 to 31 December 2022, stratified by analgesic class

**Three months interval relative to date of enrolment (index date)**									
	**Pre-enrolment**				**Enrolment**	**Post-enrolment**			
**Quarter year**	**-4**	**-3**	**-2**	**-1**	**0**	**1**	**2**	**3**	**4**
Paracetamol	16,658	17,359	18,892	23,413	27,648	25,049	23,899	24,073	23,914
NSAIDs	5,968	5,957	6,527	9,096	9,723	7,417	6,515	6,415	5,981
Opioids	4,318	4,236	4,129	5,090	6,172	4,997	4,752	4,443	4,430
Gabapentinoids	3,036	3,189	3,410	4,549	7,395	6,679	6,285	6,031	5,955
SNRIs	2,287	2,865	2,915	2,866	3,129	3,245	3,190	3,349	3,422
TCAs	656	734	682	797	995	892	877	871	859
Muscle relaxants	332	337	398	559	824	597	563	525	615
Any use	33,254	34,677	36,952	46,371	55,886	48,875	46,082	45,707	45,176

DDD, defined daily dose; NSAIDs, non-steroidal anti-inflammatory drugs; SNRIs, serotonin-norepinephrine reuptake inhibitors; TCAs, tricyclic antidepressants.

Figure S1 Quarterly DDDs of prescribed analgesics per 1,000 individuals before, during, and after a standardised chiropractic care programme, stratified by calendar year

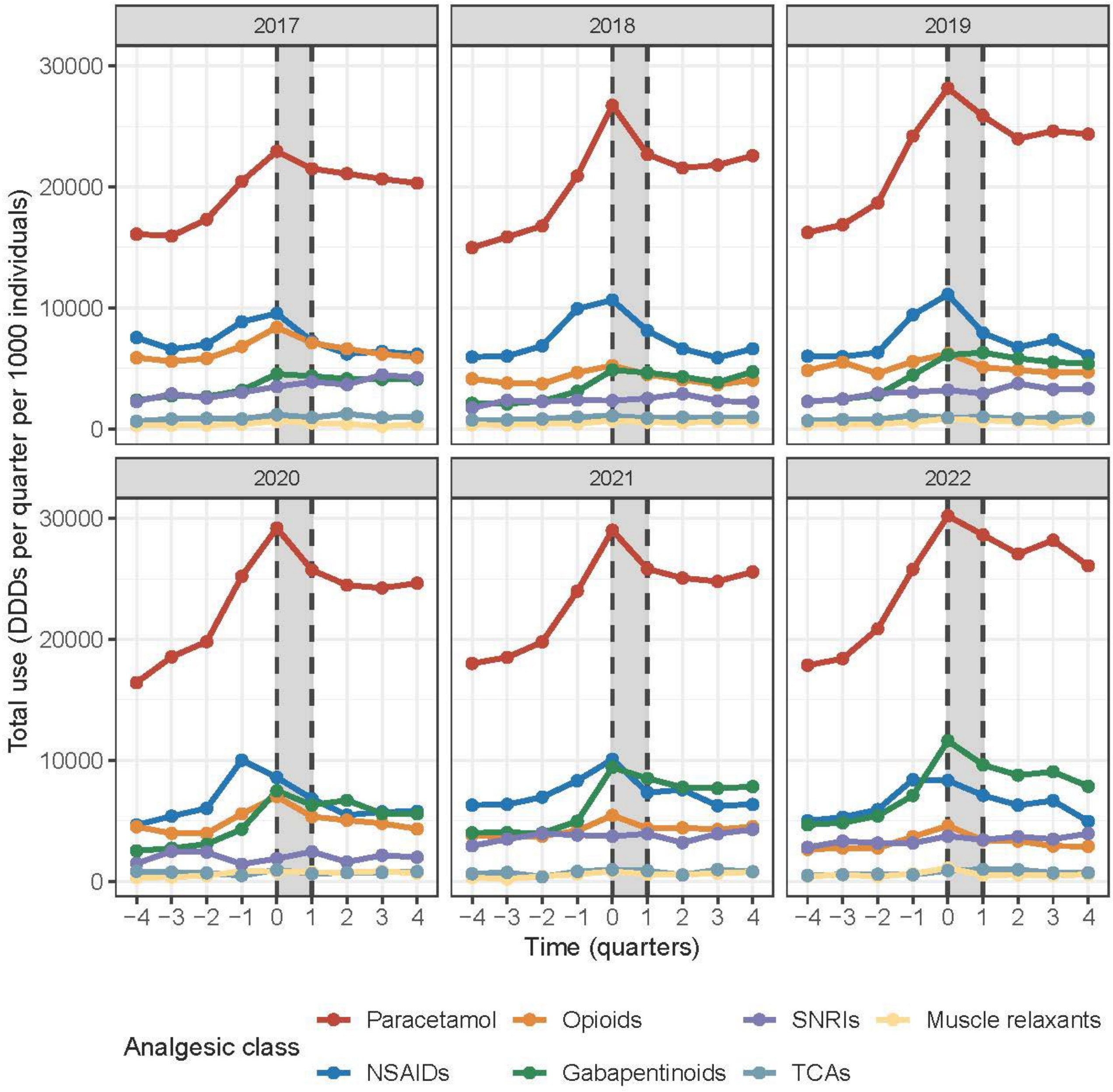

Total use of analgesics per quarter per 1,000 individuals among 7,294 patients with lumbar spinal stenosis before, during, and after enrolment in a standardised chiropractic care programme in Denmark, stratified by calendar year. Interval quarter 0 corresponds to the time of enrolment in the care programme. DDD, defined daily doses; NSAID, non-steroidal anti-inflammatory drugs; SNRIs, serotonin-norepinephrine reuptake inhibitors; TCAs, tricyclic antidepressants.
